# Recovery From COVID-19–Related Disruptions in Cancer Detection

**DOI:** 10.1001/jamanetworkopen.2024.39263

**Published:** 2024-10-14

**Authors:** Uriel Kim, Johnie Rose, Bryan T. Carroll, Richard S. Hoehn, Eric Chen, Jeremy S. Bordeaux, Siran M. Koroukian

**Affiliations:** 1 Department of Internal Medicine, Cedars-Sinai Medical Center, Los Angeles, California; 2 Case Comprehensive Cancer Center, School of Medicine, Case Western Reserve University, Cleveland, Ohio; 3 Department of Population and Quantitative Health Sciences, School of Medicine, Case Western Reserve University, Cleveland, Ohio; 4 Center for Community Health Integration, School of Medicine, Case Western Reserve University, Cleveland, Ohio; 5 Department of Dermatology, University Hospitals Cleveland Medical Center, Cleveland, Ohio; 6 Division of Surgical Oncology, University Hospitals Cleveland Medical Center, Cleveland, Ohio; 7 Department of Radiation Oncology, School of Medicine, University of California, Irvine

## Abstract

**Importance:**

The COVID-19 pandemic impacted the timely diagnosis of cancer, which persisted as the second leading cause of death in the US throughout the pandemic.

**Objective:**

To evaluate the disruption and potential recovery in cancer detection during the first (2020) and second (2021) years of the COVID-19 pandemic.

**Design, Setting, and Participants:**

This cross-sectional study involved an epidemiologic analysis of nationally representative, population-based cancer incidence data from the Surveillance, Epidemiology, and End Results (SEER) Program. Included patients were diagnosed with incident cancer from January 1, 2000, through December 31, 2021. The analysis was conducted in May 2024 using the April 2024 SEER data release, which includes incidence data through December 31, 2021.

**Exposures:**

Diagnosis of cancer during the first 2 years of the COVID-19 pandemic (2020, 2021).

**Main Outcomes and Measures:**

Difference between the expected and observed cancer incidence in 2020 compared with 2021, with additional analyses by demographic subgroups (sex, race and ethnicity, and age group) and community (county-level) characteristics.

**Results:**

The analysis included 15 831 912 patients diagnosed with invasive cancer between 2000 and 2021, including 759 810 patients in 2020 and 825 645 in 2021. The median age was 65 years (IQR, 56-75 years), and 51.0% were male. The percentage difference between the expected and observed cancer incidence was −8.6% (95% CI, −9.1% to −8.1%) in 2020, with no significant difference in 2021 (−0.2%; 95% CI, −0.7% to 0.4%). These translated to a cumulative (2020-2021) deficit in observed vs expected cases of −127 931 (95% CI, −139 206 to −116 655). Subgroup analyses revealed that incidence rates remained substantially depressed from expected rates into 2021 for patients living in the most rural counties (−4.9%; 95% CI, −6.7% to −3.1%). The cancer sites with the largest cumulative deficit in observed vs expected cases included lung and bronchus (−24 940 cases; 95% CI, −28 936 to −20 944 cases), prostate (−14 104 cases; 95% CI, −27 472 to −736 cases), and melanoma (−10 274 cases; 95% CI, −12 825 to −7724 cases).

**Conclusions and Relevance:**

This cross-sectional study of nationally representative registry data found that cancer incidence recovered meaningfully in 2021 following substantial disruptions in 2020. However, incidence rates need to recover further to address the substantial number of patients with undiagnosed cancer during the pandemic.

## Key Points

**Question:**

To what extent was cancer detection in the US disrupted during the first year of the COVID-19 pandemic, and how much did it recover in the second year?

**Findings:**

In this cross-sectional study of 15 831 912 patients diagnosed with invasive cancer between 2000 and 2021, estimated cancer incidence was 9% below projections in 2020; there was no significant difference between projected and actual incidence in 2021.

**Meaning:**

The findings suggest that cancer detection needs to recover further in future years to address patients whose cancer went undiagnosed because of the pandemic.

## Teaser

This cross-sectional study compares observed vs expected rates of cancer diagnosis in the US during the first and second years of the COVID-19 pandemic to assess disruptions to and recovery in cancer detection.

## Introduction

The COVID-19 pandemic was disruptive to the delivery of oncologic services across the entire cancer care spectrum, impacting screening,^1,2^ diagnosis,^3,4^ treatment,^5^ and survivorship.^6,7^ As an illustration of the magnitude of this disruption, cancer incidence fell by nearly 9% during the first year (2020) of the pandemic^3^ and by almost 50% during the peak lockdown months (March through May 2020).^8^

The ability of patients to receive screening and diagnostic cancer care during the pandemic reflected a multitude of factors. From a health care delivery perspective, especially during the early parts of the pandemic, access to diagnostic cancer services was likely constrained due to the reallocation of health care resources to contain, understand, and treat COVID-19.^9^ From the patient perspective, the willingness to seek cancer screening services or care for symptomatic disease during the pandemic was likely highly variable, as evidenced by the heterogenous risk tolerance regarding COVID-19 by sociodemographic groups.^10^

While the challenges posed by the pandemic for health care systems to deliver and for patients to receive health care were substantial, there were several notable adaptations that emerged. A key example was the ubiquity of telemedicine services, which prior to the pandemic had limited adoption by both patients and physicians due to the lack of infrastructure, regulatory support, and reimbursement.^11^ More specifically to cancer services, health systems generally prioritized appointments and resources for those requiring diagnostic care for suspected malignant neoplasms over competing medical concerns,^12,13^ while efforts were made to reduce the number of in-person visits and to shorten the cancer treatment durations.^14,15,16^

Thus, the effects of COVID-19 on cancer care services were likely highly dynamic. To evaluate the evolving outcomes of the COVID-19 pandemic with regard to cancer detection, we conducted an epidemiologic analysis of incidence using the latest (April 2024) release of Surveillance, Epidemiology, and End Results (SEER) Program data, which included nationally representative cancer registry data through December 31, 2021. We focused on cancer incidence during the first 2 years (2020, 2021) of the pandemic to evaluate the disruption to and potential recovery of cancer detection and diagnosis, further contextualizing the magnitude of these changes by estimating the deficit in observed cancer cases at the national level.

## Methods

The protocol for this cross-sectional study was reviewed by the institutional review board of Case Western Reserve University, which exempted the study from further review, including waiver of informed consent, because it was deemed not human participants research. The overall design, analysis, and presentation of this study was informed by the Strengthening the Reporting of Observational Studies in Epidemiology (STROBE) reporting guideline for cross-sectional studies: we thoroughly described the study setting, patient inclusion criteria, and methods of data collection; the statistical methods were replicable; and we cautiously interpreted the results, accounting for the statistical uncertainty and limitations of the dataset. The analyses were conducted in May 2024.

### Statistical Analysis

We estimated the percentage difference between the expected vs observed incidence of cancer in 2020 using SEER data. The approach is described more extensively elsewhere,^3^ but in brief, the expected incidence in the first year of the pandemic (2020) was forecasted using joinpoint trend modeling^17^ trained on 20 years of data (2000-2019) before the pandemic. The process was extended to the second year of the pandemic (2021) to evaluate the potential recovery of cancer incidence rates. We conducted these analyses for all cancer types combined, additionally stratifying by (and separately modeling) select demographic and community (county-level) characteristics and by major cancer sites. All incidence rates were age adjusted and were additionally delay adjusted^18^ when possible. Demographic information included a combined race and ethnicity variable, which was reported in 5 mutually exclusive categories: Hispanic, non-Hispanic American Indian or Alaska Native, non-Hispanic Asian or Pacific Islander, non-Hispanic Black, non-Hispanic White, and non-Hispanic unknown race. This variable was derived directly from SEER and was included in the study given the differential impacts of COVID-19 across racial and ethnic groups^19^ as well as the historic differences in access to screening and diagnostic cancer services across racial and ethnic groups.^20^

Next, the national deficit in observed cancer cases was estimated to contextualize the findings. This deficit was modeled by extrapolating the observed cancer case counts in SEER and the percentage difference estimates for 2020 and 2021 to national population-at-risk data from the National Cancer Institute and the US Census Bureau.

All tests of statistical significance were conducted at the α =  .05 level. The 95% CIs for incidence rates were calculated using the method of Tiwari et al.^21^ The 95% CIs for trend models were generated using the parametric approach,^22^ while optimal trend models were selected using the permutation test.^23^ Data from SEER were accessed via SEER*Stat, version 8.4.3 (National Cancer Institute), while the trend analyses were developed using the Joinpoint Regression Program, version 4.9.1 (National Cancer Institute).

## Results

The study included 15 831 912 patients diagnosed with malignant cancer between January 1, 2000, and December 31, 2021. The trend model was trained on patients diagnosed between 2000 and 2019 (n = 14 246 457) and was then used to project the expected incidence of cancer in 2020 and 2021. These expected rates were then compared with the observed rates of cancer in 2020 and 2021, during which there were 759 810 and 825 645 observed cases, respectively. Baseline characteristics of the patients included in the study (diagnosed with cancer in 2000-2021) and those diagnosed with cancer in the peripandemic years of 2018, 2019, 2020, and 2021 are presented in Table 1. Overall, the median age was 65 years (IQR, 56-75 years); 49.0% were female, and 51.0% were male. A total of 0.4% were American Indian or Alaska Native; 5.0%, Asian or Pacific Islander; 10.4%, Black; 11.1%, Hispanic; 72.4%, White; and 0.8%, unknown race.

**Table 1.** Tumor, Demographic, and County-Level Characteristics of Patients in SEER Between 2000 and 2021

Characteristic	Patients, No. (%)				
	2000-2021 (N = 15 831 912)	2018 (n = 804 866)	2019 (n = 830 007)	COVID-19 pandemic	
				2020 (n = 759 810)	2021 (n = 825 645)
**Patient characteristics**					
Cancer stage					
Localized	5 918 091 (44.5)^a^	362 261 (45.0)	378 254 (45.6)	336 333 (44.3)	382 086 (46.3)
Regional	2 708 895 (20.4)^a^	160 665 (20.0)	167 661 (20.2)	154 777 (20.4)	167 582 (20.3)
Distant	3 286 781 (24.7)^a^	196 180 (24.4)	202 274 (24.4)	193 860 (25.5)	202 914 (24.6)
NA	1 391 722 (10.5)^a^	85 760 (10.7)	81 818 (9.9)	74 840 (9.8)	73 063 (8.8)
Sex					
Female	7 764 848 (49.0)	397 425 (49.4)	409 082 (49.3)	373 737 (49.2)	408 989 (49.5)
Male	8 067 064 (51.0)	407 441 (50.6)	420 925 (50.7)	386 073 (50.8)	416 656 (50.5)
Race and ethnicity					
Hispanic, any race	1 760 291 (11.1)	104 910 (13.0)	110 337 (13.3)	102 187 (13.4)	114 105 (13.8)
Non-Hispanic American Indian or Alaska Native	62 240 (0.4)	3701 (0.5)	3826 (0.5)	3627 (0.5)	3989 (0.5)
Non-Hispanic Asian or Pacific Islander	793 629 (5.0)	47 768 (5.9)	49 855 (6.0)	45 327 (6.0)	52 840 (6.4)
Non-Hispanic Black	1 641 837 (10.4)	86 585 (10.8)	90 049 (10.8)	81 576 (10.7)	90 604 (11.0)
Non-Hispanic White	11 455 169 (72.4)	553 360 (68.8)	566 124 (68.2)	516 186 (67.9)	549 246 (66.5)
Non-Hispanic unknown race	118 746 (0.8)	8542 (1.1)	9816 (1.2)	10 907 (1.4)	14 861 (1.8)
Age group, y					
<20	164 093 (1.0)	7958 (1.0)	7605 (0.9)	7406 (1.0)	7396 (0.9)
20-39	748 957 (4.7)	37 632 (4.7)	38 465 (4.6)	35 448 (4.7)	37 745 (4.6)
40-64	6 226 202 (39.3)	307 318 (38.2)	312 208 (37.6)	280 728 (36.9)	302 699 (36.7)
65-79	6 101 296 (38.5)	328 113 (40.8)	344 151 (41.5)	319 726 (42.1)	353 089 (42.8)
≥80	2 591 364 (16.4)	123 845 (15.4)	127 578 (15.4)	116 502 (15.3)	124 716 (15.1)
**County characteristics**					
Rurality					
Large metropolitan	9 848 519 (62.2)	505 006 (62.7)	520 798 (62.7)	473 779 (62.4)	518 528 (62.8)
Medium metropolitan	2 804 758 (17.7)	145 788 (18.1)	150 129 (18.1)	138 757 (18.3)	150 910 (18.3)
Small metropolitan	1 226 529 (7.8)	59 504 (7.4)	60 930 (7.3)	57 166 (7.5)	60 878 (7.4)
Rural, adjacent to metropolitan area	1 251 502 (7.9)	60 384 (7.5)	62 872 (7.6)	57 982 (7.6)	61 220 (7.4)
Rural, not adjacent to metropolitan area	688 356 (4.4)	33 587 (4.2)	34 660 (4.2)	31 586 (4.2)	33 499 (4.1)
NA	12 248 (0.1)	597 (0.1)	618 (0.1)	540 (0.1)	610 (0.1)
Poverty rate, %					
<10	5 279 614 (33.3)	272 665 (33.9)	281 810 (34.0)	259 657 (34.2)	284 981 (34.5)
10-19.99	9 513 129 (60.1)	480 149 (59.7)	494 305 (59.6)	451 198 (59.4)	488 202 (59.1)
≥20	1 036 340 (6.5)	51 972 (6.5)	53 828 (6.5)	48 917 (6.4)	52 413 (6.3)
NA	2829 (<0.1)	80 (<0.1)	64 (<0.1)	38 (<0.1)	49 (<0.1)
Non-US born, %					
<10	5 363 918 (33.9)	275 841 (34.3)	283 125 (34.1)	263 267 (34.6)	282 267 (34.2)
10-19.99	3 733 414 (23.6)	193 317 (24)	200 783 (24.2)	184 986 (24.3)	200 527 (24.3)
≥20	6 731 751 (42.5)	335 628 (41.7)	346 035 (41.7)	311 519 (41.0)	342 802 (41.5)
NA	2829 (<0.1)	80 (<0.1)	64 (<0.1)	38 (<0.1)	49 (<0.1)
No high school education, %					
<10	6 272 115 (39.6)	322 782 (40.1)	332 592 (40.1)	307 676 (40.5)	336 707 (40.8)
10-19.99	8 604 235 (54.4)	434 432 (54.0)	447 033 (53.9)	406 971 (53.6)	440 989 (53.4)
≥20	952 733 (6.0)	47 572 (5.9)	50 318 (6.1)	45 125 (5.9)	47 900 (5.8)
NA	2829 (<0.1)	80 (<0.1)	64 (<0.1)	38 (<0.1)	49 (<0.1)

Abbreviations: NA, not available; SEER, Surveillance, Epidemiology, and End Results.

^a^ The stage-specific analysis includes 13 305 489 cases from 2004 onward, when the staging variable became consistently available.

The forecasted cancer incidence in 2020 and 2021 in the absence of the pandemic was 458.12 (95% CI, 456.71-459.54) and 459.06 (95% CI, 457.64-460.48) per 100 000 population, respectively (Figure). The observed incidence during the pandemic in 2020 and 2021 was 418.90 (95% CI, 417.94-419.86) and 458.33 (95% CI, 457.32-459.34) per 100 000 population, respectively. There was an estimated substantial disruption in the detection and diagnosis of cancer in 2020, with a percentage difference between the expected and observed incidence of −8.6% (95% CI, −9.1% to −8.1%), followed by a recovery in the incidence rate in 2021, as suggested by the nonsignificant percentage difference of −0.2% (95% CI, −0.7% to 0.4%).

**Figure.**
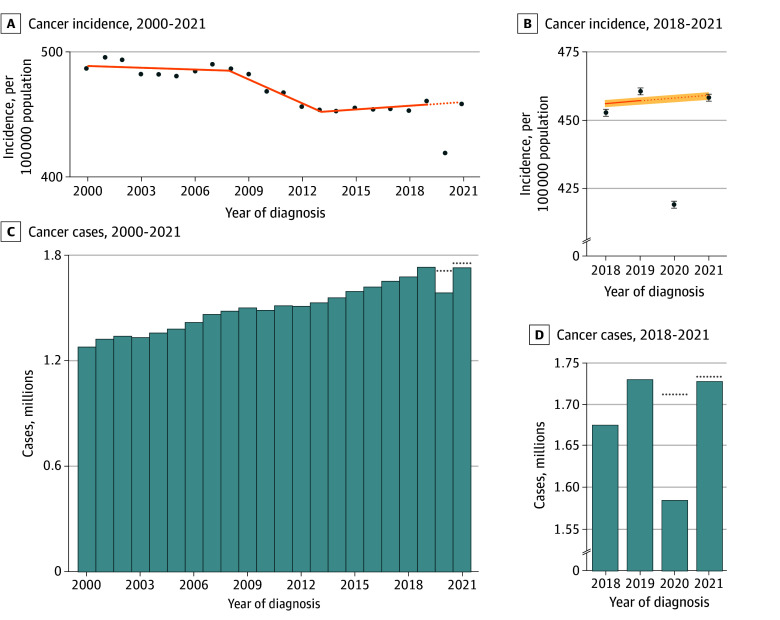
US Cancer Incidence From 2000 to 2021 and During Peripandemic Years 2018 to 2021 Observed total cancer cases in the US were extrapolated from Surveillance, Epidemiology, and End Results Program data, and cancer incidence rates were modeled using joinpoint trend modeling. A and B, Solid lines indicate modeled trend and dashed lines, projected incidence. B, Error bars indicate 95% CIs for observed incidence and shading, 95% CIs for modeled trend and projected incidence. C and D, Bars indicate observed cases and dotted line, projected cases.

Translating these findings into an estimated deficit in observed cases, there were an expected 1 712 158 cases of cancer nationally in 2020 but only 1 586 991 observed cases (Figure). This translated to a difference of −125 167 cases (95% CI, −131 771 to −118 503 cases). Although not a significant difference, the estimated deficit was substantially smaller in 2021 (−2764 cases; 95% CI, −11 833 to 6402 cases), causing the cumulative (2020-2021) estimated deficit to grow minimally to −127 931 cases (95% CI, −139 206 to −116 655 cases).

While the pandemic was broadly disruptive, subgroup analyses (Table 2) revealed variation in the impact of the pandemic by demographic characteristics and community (county-level) characteristics. During the first year (2020) of the pandemic, there was a relatively larger decrease in incidence observed for localized cancers and for male patients. With regard to community characteristics, larger decreases in incidence were observed for patients living in counties that had lower poverty rates, higher percentage of non–US-born residents, higher educational levels, or were on either extreme of the rural-urban continuum. By the second year, all the incidence rates for the subgroups had recovered (ie, 2021 percentage differences were less than 2020 percentage differences) to some degree compared with 2020. Among patient subgroups, those living in rural counties had cancer incidence rates that remained substantially depressed (eg, in 2021, the difference between expected and observed incidence was −4.9% [95% CI, −6.7% to −3.1%] in rural counties not adjacent to a metropolitan area). Conversely, incidence rates were significantly higher (ie, 2021 percentage difference >0%) for patients who were female, younger than 20 years, and Asian or Pacific Islander.

**Table 2.** Percentage Difference in Expected vs Observed Cancer Incidence by Subgroups

Subgroup	Incidence, per 100 000 population					
	Pandemic year 1 (2020)			Pandemic year 2 (2021)		
	Expected	Observed	Difference (95% CI), %	Expected	Observed	Difference (95% CI), %
**Patient characteristics**						
Cancer stage						
All	458.12	418.90	−8.6 (−9.1 to −8.1)	459.06	458.33	−0.2 (−0.7 to 0.4)
Localized	206.67	180.81	−12.5 (−14.2 to −10.8)	208.83	203.02	−2.8 (−4.6 to −1.0)
Regional	91.38	84.11	−8.0 (−8.2 to −7.7)	91.23	90.09	−1.3 (−1.5 to −1.0)
Distant	110.79	104.67	−5.5 (−6.2 to −4.9)	110.94	108.07	−2.6 (−3.2 to −1.9)
Sex						
Female	429.11	396.32	−7.6 (−8.0 to −7.3)	429.35	437.70	1.9 (1.5 to 2.4)
Male	497.10	455.46	−8.4 (−8.8 to −8.0)	497.39	493.51	−0.8 (−1.2 to −0.4)
Race and ethnicity						
Hispanic, any race	370.89	336.36	−9.3 (−10.4 to −8.2)	372.41	372.76	0.1 (−1.1 to 1.3)
Non-Hispanic American Indian or Alaska Native	465.36	438.13	−5.8 (−11.2 to −0.5)	470.31	477.87	1.6 (−4.0 to 7.2)
Non-Hispanic Asian or Pacific Islander	313.72	285.09	−9.1 (−9.6 to −8.6)	312.89	329.66	5.4 (4.9 to 5.9)
Non-Hispanic Black	474.62	427.72	−9.9 (−10.8 to −8.9)	475.66	477.41	0.4 (−0.6 to 1.4)
Non-Hispanic White	493.46	452.47	−8.3 (−9.0 to −7.6)	495.10	489.37	−1.2 (−1.9 to −0.4)
Age group, y						
<20	18.95	18.67	−1.5 (−2.6 to −0.4)	18.80	19.30	2.7 (1.5 to 3.8)
20-39	93.06	84.87	−8.8 (−11.0 to −6.6)	93.88	92.16	−1.8 (−4.1 to 0.5)
40-64	547.64	500.64	−8.6 (−9.1 to −8.1)	548.15	552.41	0.8 (0.3 to 1.3)
65-79	1984.08	1795.00	−9.5 (−10.6 to −8.4)	1995.55	1962.25	−1.7 (−2.9 to −0.5)
≥80	2285.05	2110.37	−7.6 (−7.9 to −7.4)	2280.66	2287.08	0.3 (0.0 to 0.6)
**County characteristics**						
Rurality						
Large metropolitan	444.38	400.13	−10.0 (−10.2 to −9.7)	444.35	432.18	−2.7 (−3.0 to −2.5)
Medium metropolitan	455.32	415.89	−8.7 (−9.4 to −7.9)	456.22	445.94	−2.3 (−3.1 to −1.5)
Small metropolitan	477.39	439.34	−8.0 (−9.2 to −6.7)	479.06	462.36	−3.5 (−4.8 to −2.2)
Rural, adjacent to metropolitan area	487.12	447.58	−8.1 (−9.6 to −6.6)	489.59	468.27	−4.4 (−5.9 to −2.8)
Rural, not adjacent to metropolitan area	473.36	428.41	−9.5 (−11.2 to −7.8)	475.87	452.37	−4.9 (−6.7 to −3.1)
Poverty rate, %						
<10	455.97	414.61	−9.1 (−9.3 to −8.8)	455.59	447.06	−1.9 (−2.1 to −1.6)
10-19.99	449.37	407.71	−9.3 (−9.6 to −8.9)	449.54	436.48	−2.9 (−3.2 to −2.6)
≥20	441.77	407.85	−7.7 (−8.1 to −7.2)	440.48	426.12	−1.0 (−1.4 to −0.6)
Non-US born, %						
<10	492.29	449.94	−8.6 (−9.6 to −7.6)	494.58	475.86	−3.8 (−4.8 to −2.7)
10-19.99	458.31	418.06	−8.8 (−9.2 to −8.4)	458.20	446.03	−2.7 (−3.1 to −2.3)
≥20	420.83	377.81	−10.2 (−10.3 to −10.2)	420.02	411.44	−2.0 (−2.1 to 2.0)
No high school education, %						
<10	476.78	433.55	−9.1 (−9.6 to −8.6)	477.32	466.27	−2.3 (−2.8 to −1.8)
10-19.99	438.42	396.64	−9.5 (−9.8 to −9.3)	438.33	425.64	−2.9 (−3.2 to −2.6)
≥20	415.76	383.89	−7.7 (−8.0 to −7.4)	414.03	405.18	−2.1 (−2.4 to −1.9)

Table 3 presents percentage difference data and the estimated deficit in diagnosed cases by select cancer sites. Cancer sites that continued to have depressed incidence rates in 2021 similar in magnitude to those from 2020 included gallbladder, eye, and orbit. Sites that showed recovery in incidence rates in 2021 but still had statistically significantly lower rates compared with projections included the larynx, lung and bronchus, soft tissue (including heart), kidney and renal pelvis, and thyroid. Cancer sites that had a full recovery of cancer incidence rates in excess of 2021 projections, leading to a cumulative surplus of expected cases, included the stomach (1516 cases; 95% CI, 1150-1882 cases) and chronic lymphocytic leukemia (643 cases; 95% CI, 348-939 cases). The cancer sites with the largest cumulative (2020-2021) deficit in expected cases included the lung and bronchus (−24 940 cases; 95% CI, −28 936 to −20 944 cases), prostate (−14 104 cases; 95% CI, −27 472 to −736 cases), and melanoma (−10 274 cases; 95% CI, −12 825 to −7724 cases).

**Table 3.** Cumulative Deficit or Surplus of Observed Cases Compared With Expected Cases by Cancer Site

Cancer site	Pandemic year 1 (2020)				Pandemic year 2 (2021)				Cumulative difference in observed vs expected cases, No. (95% CI)
	Observed cases		Observed vs expected cases		Observed cases		Observed vs expected cases		
	Incidence, per 100 000 population	US estimate, No.	Incidence, %^a^	No.	Incidence, per 100 000 population	US estimate, No.	Incidence, %^a^	No.	
All	418.90	1 586 991	−8.6	−125 167	458.33	1 729 652	−0.2	−2764	−127 931 (−139 206 to −116 655)
Oral cavity and pharynx	11.13	43 440	−6.0	−2446	11.87	46 237	−0.4	−180	−2626 (−3995 to −1258)
Tongue	3.57	14 124	−7.7	−1006	3.81	15 088	−3.3	−483	−1489 (−2406 to −571)
Tonsil and oropharynx	2.82	11 298	−4.7	−508	2.96	11 868	−2.4	−282	−790 (−1775 to 194)
Digestive system									
Esophagus	4.07	16 237	−4.1	−636	4.21	16 812	−0.7	−115	−751 (−1185 to −318)
Stomach	6.49	24 665	−8.0	−1830	7.72	29 524	10.2	3346	1516 (1150 to 1882)
Small intestine	2.45	9169	−8.2	−695	2.87	10 607	5.6	625	−70 (−849 to 709)
Colon and rectum	33.65	126 575	−9.4	−10 875	37.49	140 925	1.9	2680	−8195 (−9195 to −7196)
Colon	23.34	87 812	−9.6	−7657	25.86	97 348	1.4	1341	−6316 (−7240 to −5392)
Rectum	10.30	38 764	−8.9	−3175	11.64	43 576	3.3	1486	−1690 (−2150 to −1229)
Anus and anorectum	1.93	7528	−7.3	−512	2.03	7902	−4.2	−321	−833 (−1385 to −281)
Liver and intrahepatic bile duct	9.00	35 896	−8.4	−2774	9.72	37 953	−1.6	−599	−3373 (−4418 to −2328)
Gallbladder	1.17	4482	−5.4	−230	1.15	4433	−7.1	−294	−524 (−747 to −301)
Pancreas	13.38	52 045	−4.9	−2411	13.92	53 996	−2.3	−1224	−3635 (−5824 to −1445)
Larynx	2.40	9677	−9.8	−861	2.50	10 114	−4.5	−435	−1296 (−1364 to −1228)
Lung and bronchus	45.25	178 251	−10.0	−16 148	46.93	185 339	−5.0	−8792	−24 940 (−28 936 to −20 944)
Bones and joints	0.98	3317	−5.7	−180	1.08	3547	2.2	79	−100 (−394 to 193)
Soft tissue, including heart	3.32	11 864	−6.7	−746	3.45	12 243	−3.5	−413	−1159 (−1678 to −640)
Melanoma	19.11	71 441	−14.8	−9210	22.40	83 685	−1.3	−1064	−10 274 (−12 825 to −7724)
Breast	122.03	237 355	−7.6	−16 860	137.42	268 719	3.5	9671	−7190 (−11 886 to −2494)
Genital system									
Cervix	7.12	12 147	−8.0	−895	7.75	12 997	0.3	41	−853 (−1261 to −446)
Uterus	26.68	55 058	−8.9	−4483	29.37	60 684	−1.0	−606	−5089 (−7434 to −2744)
Ovary	9.73	18 863	−6.4	−1133	10.76	20 626	5.2	1131	−2 (−113 to 109)
Vulva	2.43	4892	−7.8	−355	2.74	5538	3.1	178	−178 (−564 to 209)
Vagina	0.64	1297	−3.0	−38	0.67	1378	1.9	27	−11 (−142 to 120)
Prostate	110.56	206 247	−9.8	−18 363	127.97	237 429	1.8	4259	−14 104 (−27 472 to −736)
Testis	5.96	9614	−1.1	−106	6.06	9674	−0.1	−13	−119 (−652 to 415)
Urinary system									
Bladder	17.44	66 879	−5.6	−3522	18.22	70 219	−0.3	−189	−3710 (−4194 to −3226)
Kidney and renal pelvis	16.58	62 583	−9.9	−5659	17.74	66 281	−5.0	−3147	−8807 (−11 206 to −6407)
Eye and orbit	0.79	2849	−7.8	−206	0.79	2857	−8.3	−218	−424 (−590 to −258)
Brain and other nervous system	6.20	22 209	−2.0	−429	6.11	21 552	−3.1	−643	−1072 (−1458 to −685)
Thyroid	12.07	41 675	−16.7	−5972	13.62	46 643	−6.5	−2861	−8833 (−9880 to −7786)
Hodgkin lymphoma	2.44	8250	−1.7	−137	2.49	8340	1.3	107	−30 (−211 to 150)
Non-Hodgkin lymphoma	17.96	67 146	−6.4	−4012	18.96	70 642	−0.7	−523	−4535 (−4982 to −4089)
Myeloma	7.23	26 580	−7.7	−1904	7.69	27 869	−3.0	−802	−2706 (−3891 to −1521)
Leukemia									
Any	14.43	49 965	−4.8	−2293	15.44	52 048	1.6	867	−1426 (−2496 to −356)
Acute lymphocytic	1.86	5840	−0.7	−42	1.88	5818	−0.4	−23	−65 (−487 to 356)
Chronic lymphocytic	4.89	16 688	−4.7	−745	5.38	17 585	7.3	1389	643 (348 to 939)
Acute myeloid	4.14	15 247	−2.3	−336	4.31	15 787	1.9	298	−38 (−552 to 476)
Chronic myeloid	2.02	6880	−6.9	−443	2.25	7429	2.3	177	−266 (−807 to 275)

^a^ The 95% CIs for the percentage differences are shown in Table 2.

## Discussion

Our analysis revealed that cancer detection and diagnosis improved broadly in 2021 after substantial disruptions in 2020. However, there was significant heterogeneity in the degree of recovery by patient demographic characteristics, community characteristics, and cancer site, and 127 931 patients were estimated to have undiagnosed cancer due to disruptions caused by the pandemic.

The substantial number of patients with undiagnosed cancer emphasizes the need for continued epidemiologic surveillance. As of 2021, most cancer sites had incidence rates that were close to baseline while having a cumulative (2020-2021) deficit in diagnosed cases, suggesting that incidence rates need to continue to improve further for several years to reach patients missed during the pandemic. Only 2 sites (stomach and chronic lymphocytic leukemia) had cancer incidence rates in excess of projections (and a statistically significant positive surplus in cumulative expected cases), suggesting that any potential patients missed during the pandemic have likely received a diagnosis of cancer. Thus, as subsequent years of data become available, it will be essential to evaluate whether cancer incidence rates improve further commensurate to the number of patients with undiagnosed cancer for sites other than stomach and chronic lymphocytic leukemia. If cancer incidence rates do not improve further, a surge of patients with more advanced disease may be anticipated in future years. An opportunity to mitigate the negative impacts of the pandemic would be to reestablish cancer screening regimens (such as for breast, cervical, lung, and colorectal cancers), which continued to have lower rates than prepandemic levels into the second year (2021) of the pandemic.^24^

The timely detection and diagnosis of cancer during the pandemic reflect a complex function of not only the adequate capacity of health care systems to deliver cancer care^15^ but also patients’ willingness and ability to seek it. Variations in patient willingness to seek diagnostic cancer care could explain some of the observed differences in the decreases in cancer incidence rates during the first year of the pandemic. For example, the finding that counties with a lower poverty rate experienced a relatively larger decrease in incidence in 2020 may be reconciled by previous observations that individuals with higher income practiced a greater degree of self-protective behaviors against COVID-19, such as handwashing and social distancing,^10^ which could translate to a reduced willingness to seek nonemergent medical care. Similarly, patients living in urban counties may have been less willing to seek care since the perceived and actual threat of COVID-19 transmission and infection was higher in more densely populated areas.^25^ However, persistently lower incidence rates into 2021 and beyond more likely reflect patients’ compromised ability to receive cancer care. For example, in 2021, patients living in rural counties experienced a less robust recovery in incidence rates, which could capture the challenges of accessing both primary and specialized health care services in rural settings.^26^ Monitoring subpopulations with persistently depressed incidence rates will be key to evaluating how the COVID-19 pandemic might have exacerbated existing disparities in cancer outcomes.

### Strengths and Limitations

A key strength of this study was the use of data from SEER, which is regarded as the gold standard population-based cancer registry in the US, covering almost 50% of the US population.^27^ The SEER data were coupled with joinpoint trend modeling–based techniques to differentiate preexisting trends in cancer incidence from changes in incidence associated with the pandemic. Additionally, the approach is easily reproducible, facilitating the continued monitoring of these epidemiologic trends.

This study also has limitations. The analysis would have been improved by using a unified, high-quality, national cancer registry covering 100% of the population, although such a registry does not exist in the US. The lack of such a registry was likely to most impact the estimates of the number of cancer cases and corresponding deficits in diagnosed cases since these figures were extrapolated to the US population using analyses derived from the SEER data. This extrapolation process led to national cancer case estimates that were compatible with, albeit modestly (approximately 6%) lower than, estimates from other widely referenced estimates of annual cancer cases from the American Cancer Society^28^ and the Centers for Disease Control and Prevention US Cancer Statistics^29^ (eTable in Supplement 1).

## Conclusions

This cross-sectional study of nationally representative cancer registry data from the SEER Program found that incidence of cancer detection recovered meaningfully in 2021 following substantial disruptions in 2020. However, 127 931 patients were estimated to have undiagnosed cases nationally during the pandemic in 2021, which could have profound, long-lasting impacts. Thus, it remains essential to rapidly reestablish care for patients with undiagnosed cancer during the pandemic to prevent the exacerbation of disparities in cancer outcomes and increased cancer-related morbidity and mortality in future years.

## Notes

Supplement 1. **eTable.** Comparison of Annual Cancer Case Counts Using SEER vs Other Estimates

Supplement 2.
Data Sharing Statement
