# Supplementary material for: Recovery From COVID-19–Related Disruptions in Cancer Detection
Source: JAMA Netw Open. 2024 Oct 14;7(10):e2439263. doi: 10.1001/jamanetworkopen.2024.39263 (PMC11474412; doi:10.1001/jamanetworkopen.2024.39263)
Supplement: Supplement 1. — eTable. Comparison of Annual Cancer Case Counts Using SEER vs Other Estimates [file jamanetwopen-e2439263-s001.pdf]

Supplementary Online Content

Kim U, Rose R, Carroll BT, et al. Recovery from COVID-19–related disruptions in cancer detection. *JAMA Netw Open*. 2024;7(10):e2439263. doi:10.1001/jamanetworkopen.2024.39263

eTable. Comparison of Annual Cancer Case Counts Using SEER vs Other Estimates

This supplementary material has been provided by the authors to give readers additional information about their work.

© 2024 Kim U et al. *JAMA Network Open*.

eTable. Comparison of Annual Cancer Case Counts Using SEER vs Other Estimates

	Cancer Cases in SEER	SEER Population	US Population	Observed Annual Cancer Cases in US			% Difference in Estimates		
				SEER Estimate	CDC (USCS) Estimate	ACS Estimate	SEER VS CDC (USCS)	SEER VS ACS	CDC (USCS) vs ACS
**2010**	711,549	147,945,689	309,382,247	1,487,983	1,577,485	1,529,560	-6.0%	-2.8%	3.0%
**2011**	724,919	149,371,816	311,860,624	1,513,496	1,613,893	1,596,670	-6.6%	-5.5%	1.1%
**2012**	724,005	150,747,875	314,371,446	1,509,849	1,603,836	1,638,910	-6.2%	-8.5%	-2.2%
**2013**	734,478	152,027,804	316,765,048	1,530,358	1,636,789	1,660,290	-7.0%	-8.5%	-1.4%
**2014**	748,089	153,373,927	319,294,716	1,557,376	1,663,263	1,665,540	-6.8%	-6.9%	-0.1%
**2015**	766,090	154,692,172	321,850,521	1,593,917	1,705,334	1,658,370	-7.0%	-4.0%	2.8%
**2016**	778,528	155,883,886	324,377,907	1,620,035	1,730,525	1,685,210	-6.8%	-4.0%	2.6%
**2017**	793,844	156,890,294	326,611,185	1,652,609	1,760,488	1,688,780	-6.5%	-2.2%	4.1%
**2018**	804,866	157,676,486	328,525,933	1,676,974	1,780,222	1,735,350	-6.2%	-3.5%	2.5%
**2019**	830,007	158,338,303	330,222,008	1,731,019	1,818,739	1,762,450	-5.1%	-1.8%	3.1%
**2020**	759,810	158,719,133	331,511,512	1,586,991	1,603,844	1,806,590**^**	-1.1%	-13.8%**^**	-12.6%**^**
**2021**	825,645	158,494,424	332,031,554	1,729,652	-NA-	1,898,160	-NA-	-9.7%	-NA-
					Average percent difference (absolute):		**5.9%**	**6.0%**	**3.2%**

^ The ACS annual cancer case estimates are model-derived. The 2020 model was not adjusted for disruptions in cancer detection during the pandemic

© 2024 Kim U et al. *JAMA Network Open*.
