# Supplementary material for: Recovery From COVID-19–Related Disruptions in Cancer Detection
Source: JAMA Netw Open. 2024 Oct 14;7(10):e2439263. doi: 10.1001/jamanetworkopen.2024.39263 (PMC11474412; doi:10.1001/jamanetworkopen.2024.39263)
Supplement: Supplement 2. — Data Sharing Statement [file jamanetwopen-e2439263-s002.pdf]

Data Sharing Statement

Kim. Recovery From COVID-19–Related Disruptions in Cancer Detection. *JAMA Netw Open*. Published October 14, 2024. doi:10.1001/jamanetworkopen.2024.39263

Data
Data available: Yes
Data types: Deidentified participant data
How to access data: All data access is moderated directly by SEER
When available: With publication

Supporting Documents
Document types: None

Additional Information
Who can access the data: All data access is moderated directly by SEER
Types of analyses: All data access is moderated directly by SEER
Mechanisms of data availability: All data access is moderated directly by SEER
